# Quantification of Epistemic Capacity and Physical Frailty in Chronic Kidney Disease: Koch’s Disease Co-infection

**DOI:** 10.7759/cureus.39290

**Published:** 2023-05-21

**Authors:** Aakankshya Tripathy, Trupti R Swain, Kali P Swain, Manoranjan Pattnaik, Jyoti Prakash Sahoo

**Affiliations:** 1 Pharmacology, Srirama Chandra Bhanja Medical College and Hospital, Cuttack, IND; 2 Neurology, Srirama Chandra Bhanja Medical College and Hospital, Cuttack, IND; 3 Pulmonary Medicine, Srirama Chandra Bhanja Medical College and Hospital, Cuttack, IND; 4 Pharmacology, Kalinga Institute of Medical Sciences, Bhubaneswar, IND

**Keywords:** quality of life, chronic kidney failure, active pulmonary tuberculosis, mini-mental state examination, mental cognition

## Abstract

Background

Chronic kidney disease (CKD) and tuberculosis (TB) co-infection devastates the affected individual physically and psychologically. Moreover, poor immune status and mental turmoil worsen cognition and quality of life. Hence, studying the cognitive function and quality of life among such patients is necessary. This study aimed to determine the changes in mini-mental state examination (MMSE) score and general health questionnaire (GHQ-12) score at six months from baseline.

Methodology

This prospective, observational study was conducted at Sriram Chandra Bhanja Medical College and Hospital, India, from February 2020 to December 2021. A total of 40 patients with stage 3-4 CKD and pulmonary TB were assessed with MMSE and GHQ-12 scales at baseline, two, and six months. The study population was grouped as ≤50 and >50 years of age. We used R software (version 4.1.1) for data analysis.

Results

In total, 40 (69%) of the 58 enrolled participants completed this study. The mean age of the study population was 50.93 ± 9.83 years. The baseline MMSE scores (≤50 years: 20.8 ± 2.1, >50 years: 20.1 ± 1.7, p = 0.17) were increased (≤50 years: 25.4 ± 1.8, >50 years: 22.4 ± 1.6, p = 0.08) at six months. The baseline GHQ-12 scores (≤50 years: 22.8 ± 2.6, >50 years: 23.1 ± 2.8, p = 0.56) were reduced (≤50 years: 17.9 ± 1.9, >50 years: 20.3 ± 2.3, p = 0.14) at six months.

Conclusions

The study participants’ cognitive function and quality of life improved after six months of modified antitubercular drugs. Nevertheless, the intergroup differences were not statistically significant.

## Introduction

Immunocompromised states such as tuberculosis (TB)-chronic kidney disease (CKD) co-infection are surfacing more often than before in developing TB [1]. In India, the incidence of pulmonary TB in CKD patients is 10.5% [2]. Poor compliance in the Indian subcontinent accounts for the worsening of the disease and a long drawn-out treatment course [3]. The cognition and memory, psychological, and physical aspects of the quality of life of these patients deteriorate at an alarming rate [4,5]. Some hemodialysis patients develop dementia, anxiety, depression, and suicidal tendencies [6]. Furthermore, kidney function and cognitive power decrease considerably after 50 years of age [7,8].

TB-CKD co-infection is one of the significant health issues in developing countries. Cognitive impairment and poor health-related quality of life encumber the country’s economic growth. Some studies [9-14] have been conducted to evaluate the intellectual ability and/or quality of life in patients with either CKD or TB. So far, no such studies have been conducted among patients with CKD-TB co-infection. Therefore, we conducted this study on the cognitive function and quality of life in CKD patients with newly diagnosed pulmonary TB using a mini-mental state examination (MMSE) scale [15] and a general health questionnaire (GHQ-12) [16], respectively.

## Materials and methods

This prospective, observational study was conducted from February 2020 to December 2021 at Sriram Chandra Bhanja (SCB) Medical College, Cuttack. We obtained approval (IEC application number: 136 dated 07.02.2020) from the Institutional Ethics Committee of SCB Medical College, Cuttack, Odisha, before study initiation. Consent from the participants was obtained before enrolment. Adult patients of both sexes with known CKD evidenced by baseline estimated glomerular filtration rate (eGFR) <60 mL/minute/m^2^ and a recent diagnosis of pulmonary TB confirmed by a positive sputum smear were included in the study. All recruited patients were initially hospitalized for seven to 21 days and were provided with a modified antitubercular treatment (ATT) regimen per their age and eGFR. Individuals with extrapulmonary TB; chronic liver disease; any cardiovascular, metabolic, or endocrine disorders; end-stage renal disease (ESRD) with a documented eGFR ≤15 mL/minute/m^2^; pregnant or lactating women; renal transplant recipients; and who were not willing to give consent were excluded from the study. The primary objective was to determine the change in MMSE score at six months from baseline. The secondary objective was to determine the change in GHQ-12 score at six months from baseline.

We examined the outpatient case sheets of patients with CKD-TB. Eligible participants were divided into groups A and B, including those aged ≤50 and >50 years, respectively. The doses of pyrazinamide and ethambutol under the ATT regimen were modified according to the eGFR of the participant. The ATT regimen was monitored and titrated only by the pulmonologist. We assessed MMSE and GHQ-12 scores at the baseline visit and at two and six months. MMSE [15] is one of the most widely accepted tools for assessing cognitive function. It is an 11-question measure that evaluates five domains of cognitive function, namely, orientation, registration, attention and calculation, recall, and language. The score ranges from 0 to 30. Higher scores denote better cognitive function. A score of 23 or lower is indicative of cognitive impairment. GHQ-12 [16] is one of the most widely used tools for assessing quality of life. This shortened version of the original 28-item questionnaire contains 12 questions. Each question has four responses with corresponding scores of 0 to 3. The total added score ranges from 0 to 36. Lower scores imply better quality of life. A score of 23 or higher is indicative of poor quality of life.

We applied the Shapiro-Wilk test to check the normality of data distribution. The categorical and continuous variables were expressed as frequency (%) and mean ± standard deviation (SD). An unpaired t-test was performed to analyze the primary and secondary objectives. For the statistical analysis of the data and generation of plots, we used the R software (version 4.1.1) [17].

## Results

We screened 72 patients with CKD and newly diagnosed pulmonary TB for our study. Eleven patients had ESRD, one had colon cancer, and two did not give consent for participation. The remaining 58 were enrolled. Eighteen patients did not come for regular follow-up visits. Finally, 40 participants completed the study and were analyzed. The baseline demographic and clinical variables of the study population are shown in Table 1. The baseline characteristics of participants of both groups were similar.

**Table 1:** Baseline sociodemographic and clinical parameters of the study population. The categorical and continuous data were expressed as n (%) and mean ± standard deviation. BMI: body mass index; eGFR: estimated glomerular filtration rate; MMSE: mini-mental state examination; GHQ-12: general health questionnaire

	Total (n = 40)	Group A (n = 20)	Group B (n = 20)	P-value
Age (years)	50.93 ± 9.83	41.70 ± 3.18	60.15 ± 3.03	<0.001
Male, n (%)	20 (50%)	10 (50%)	10 (50%)	1
Weight (kg)	39.37 ± 4.24	39.56 ± 3.67	38.68 ± 4.07	0.61
BMI (kg/m^2^)	17.53 ± 1.31	17.36 ± 1.76	17.72 ± 0.98	0.84
Serum creatinine (mg/dL)	2.06 ± 0.29	2.11 ± 0.23	2.03 ± 0.19	0.53
eGFR (mL/minute/m^2^)	34.92 ± 4.17	35.95 ± 3.96	33.28 ± 4.02	0.39
MMSE score	20.5 ± 1.8	20.8 ± 2.1	20.1 ± 1.7	0.17
GHQ-12 score	23.0 ± 2.9	22.8 ± 2.6	23.1 ± 2.8	0.56

**Figure 1:**
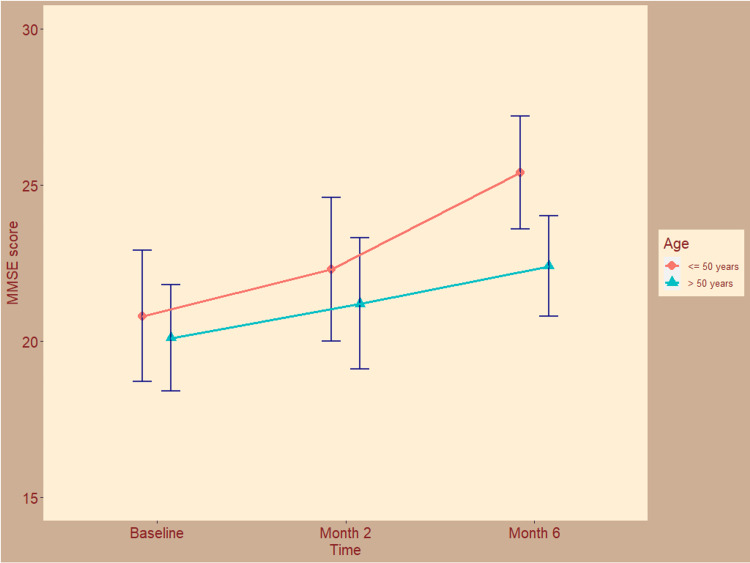
The cognitive function of the study participants assessed with the MMSE scale. The line plots with error bars show the mean mini-mental state examination (MMSE) scores. The MMSE scores range from 0 to 30. Higher scores indicate better cognitive function.

The cognitive function of the study participants assessed with the MMSE scale is shown in Figure 1. At the baseline visit, MMSE scores for both groups were 20.8 ± 2.1 and 20.1 ± 1.7, respectively (p = 0.17). After two months of the modified ATT regimen, the scores increased to 22.3 ± 2.3 and 21.2 ± 2.1, respectively (p = 0.19). After six months, the MMSE scores were 25.4 ± 1.8 and 22.4 ± 1.6, respectively (p = 0.08). The mean changes from baseline were 4.7 (95% confidence interval (CI) = 3.8 to 5.5) and 2.4 (95% CI = 1.9 to 2.9), respectively (p = 0.02), suggesting a statistically significant difference. The cognitive improvement was pronounced in younger patients.

**Figure 2:**
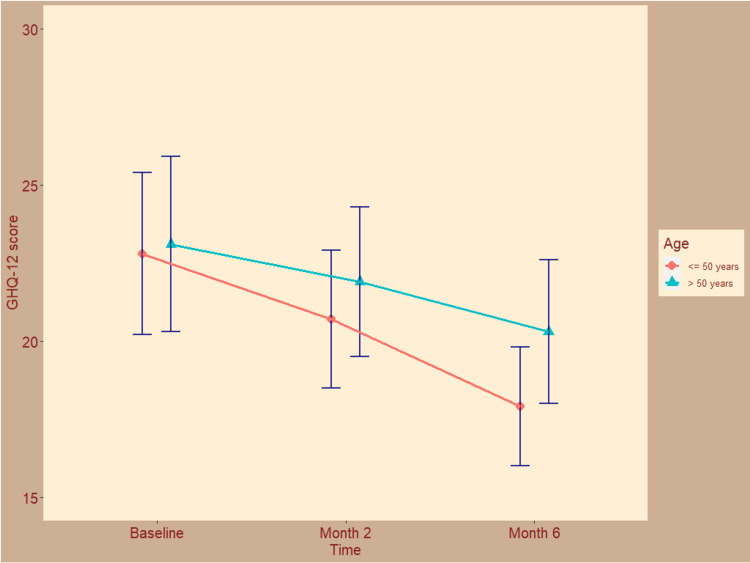
The quality of life of the study participants assessed with the GHQ-12 scale. The line plots with error bars show the mean general health questionnaire (GHQ-12) scores. The GHQ-12 scores range from 0 to 36. Lower scores indicate better quality of life.

The quality of life of the study participants assessed with the GHQ-12 scale is shown in Figure 2. At the baseline visit, GHQ-12 scores for both groups were 22.8 ± 2.6 and 23.1 ± 2.8, respectively (p = 0.56). After two months of the modified ATT regimen, the scores decreased to 20.7 ± 2.2 and 21.9 ± 2.4, respectively (p = 0.33). After six months, the GHQ-12 scores were 17.9 ± 1.9 and 20.3 ± 2.3, respectively (p = 0.14). The mean changes from baseline were -4.8 (95% CI = -5.7 to -3.9) and -2.7 (95% CI = -3.4 to -1.9), respectively (p = 0.04), suggesting a statistically significant difference. The improvement in quality of life was conspicuous in younger patients.

## Discussion

This prospective, observational study focused on the effect of modified ATT on cognitive function and quality of life among patients with CKD and pulmonary tuberculosis. For the entire study duration, all participants received modified ATT according to their renal function and age. We evaluated the changes in the MMSE [15] score and GHQ-12 [16] score after six months of treatment. We found that all the participants had improved cognitive function and quality of life. However, the more prominent effects were observed in younger participants.

The MMSE scores improved with time in both groups. The systematic review by Shen et al. [14] suggested that CKD worsens cognitive function over time. Recent studies by Martini et al. [9] and Miyazawa et al. [10] argued that antitubercular drugs improve patients’ memory and cognitive function. Our study findings concord with the studies mentioned earlier in this regard. The GHQ-12 scores lowered with time in both groups indicating better quality of life. Shen et al. [14] suggested that CKD hampers the psychological, physical, social, and economic aspects of a patients’ quality of life over time. Martini et al. [9] also reported that co-infections with TB drastically affect patients’ and their near and dear ones’ quality of life. Our study findings were consistent with these studies.

This is the first study to evaluate cognition and quality of life in patients with CKD-TB co-infection. However, the findings of this study should be analyzed with a few limitations. First, a higher attrition rate, possibly because of the pandemic, reduced the study population. Second, excluding patients with dementia, depression, and suicidal tendencies could have created recall bias. Third, we could not obtain extensive data regarding comorbidities and other concomitant medications. The renoprotective drugs contributed to the improvement of renal function. We did not analyze the contribution of those drugs to quality of life and cognition.

## Conclusions

Both cognitive function and quality of life improved at six months in patients with CKD and pulmonary TB treated with modified ATT. More studies with a larger study population are warranted, including all stages of CKD.
